# Expanding Global Rehabilitation Services through International Academic-Community Partnerships

**DOI:** 10.5334/aogh.2876

**Published:** 2020-07-01

**Authors:** Rawan AlHeresh, Peter S. Cahn

**Affiliations:** 1 “Toward an All-Inclusive Jordan”, Department of Occupational Therapy, School of Health and Rehabilitation Sciences, MGH Institute of Health Professions, Boston, MA, US; 2 Center for Interprofessional Studies and Innovation, MGH Institute of Health Professions, Boston, MA, US

## Abstract

**Background:**

More than one billion people worldwide live with a disability, yet rehabilitation professionals are scarce in low- and middle-income countries. Attempts to expand access to rehabilitation services have encountered barriers on multiple levels: limited resources on the systemic level, hierarchies on the professional level, and cultural stigma on the community level.

**Objectives:**

We sought to determine if an academic-community partnership could overcome multiple levels of barriers to expand services for people with disabilities.

**Methods:**

Toward an All-Inclusive Jordan incorporates community-based rehabilitation with prelicensure health professions education to address the three primary levels of barriers to rehabilitation services in low- and middle-income countries. The yearlong curriculum includes formal training, research, and advocacy with graduate students from the United States and health professions students and community members in Palestinian refugee camps near Amman, Jordan.

**Findings:**

After two cycles of the program, 14 Jordanian volunteers have partnered with 20 graduate students from the United States. They have delivered over 300 direct rehabilitation sessions, conducted ten workshops with mothers of children with disabilities, and trained 12 community-based rehabilitation workers in the refugee camps.

**Conclusions:**

The academic-community partnership model builds on the evidence base for the success of community-based rehabilitation services in low- and middle-income countries. Its components address barriers on multiple levels to create a sustainable expansion of services to people with disabilities.

## Background

Several recent international reports on the quality of health care have identified the shortcomings of health care systems globally, particularly in low- and middle-income countries (LMIC). In an editorial in *Archives of Physical Medicine and Rehabilitation*, Jesus and Hoenig noticed that, in discussing the need for quality improvement, the consensus documents included no mention of rehabilitation services [1]. Overlooking rehabilitation in proposals for improving health care outcomes ignores the more than one billion people worldwide living with a disability [2]. To address this gap and to enhance quality of life for people living with a disability, the authors suggest addressing the structural barriers to delivering rehabilitation services.

The 2015 public health emergency in Brazil sparked by the Zika virus illustrated the danger of focusing health care attention primarily on the immediate response without considering access to rehabilitation. When the link between congenital developmental conditions and the Zika virus was established, international research on diagnosis and treatment ramped up to supplement local efforts to contain the mosquito vector [3]. By 2017, public attention to the Zika virus had faded alongside the end of the epidemic’s acute phase [4]. Yet, for Brazilian families affected by the Zika virus, the challenge of accessing health services for children with a constellation of cognitive and physical impairments continued. Interviews with mothers by Brazilian and British researchers revealed the difficulties that parents faced in seeking ongoing care for their children [5]. The location of specialized health care institutions far from the communities most affected by the virus strained families’ budgets and lessened chances for attending rehabilitation follow-up appointments.

Of the over one billion people living with a disability, 80% live in a LMIC like Brazil, where access to rehabilitation services is severely limited [6]. Depending on their condition, people living with a disability could experience higher quality of life through rehabilitation services like physical, occupational, and speech therapy [7]. Despite the high demand for rehabilitation services, there is a dearth of health professionals qualified to deliver the services. The gap between demand and supply is particularly glaring in LMIC, where the ratio of rehabilitation professionals falls below ten per million people [7]. Workforce capacity suffers from a lack of training programs, professional recognition, specialized equipment, and government support [8]. As a result, only 3% of people in LMIC who need rehabilitation services receive them [9].

Some academics and nongovernmental organizations have recognized the urgent need to expand access to rehabilitation services for the large and growing population of people with disabilities [10]. Their recommendations tend to focus on large-scale changes to policy and health care systems. While necessary, structural changes may not arrive soon enough for the vast population of people living with disabilities. This paper presents one model of how a single global health educator can contribute to scaling up the provision of rehabilitation services in a LMIC. The initial success of Toward an All-Inclusive Jordan, a community-based rehabilitation project, suggests an alternative that may form part of a multipronged approach to enhance wellbeing for people with disabilities in every country.

## Obstacles to Rehabilitation Access

Any effort to expand access to rehabilitative services in LMIC must address obstacles on three levels: macrosystemic, professional, and community. At the macrosystemic level, the most obvious and pernicious obstacle is the competition for scarce resources. As with other health interventions that require long-term investment, rehabilitation services appear less attractive than measures that produce immediate results [11]. The primary care health needs of populations in LMIC may be so great that attending to people with chronic disabilities feels like a luxury. The case for directing resources toward rehabilitation becomes more difficult in the absence of high-quality impact evaluation studies of rehabilitation interventions for people in LMIC [12].

Another barrier to expanding rehabilitative services emerges from embedded hierarchies among the health professions. Physician dominance over health care in many LMIC may lead them to deprioritize rehabilitation, which is traditionally delivered by therapists. In high-income countries, bureaucratization and corporatization of health care has introduced outside administration that erodes physician dominance [13]. By contrast, health care systems in LMIC lack the integration and specialization to connect medical and rehabilitative services. The tilt in favor of medicine across LMIC is evident in the paucity of rehabilitation professionals, lack of knowledge of their skills, and absence of an interprofessional team approach [9].

The third level of impediment exists among communities. In many LMIC, cultural stigma against people with disabilities discourages them from seeking treatment. People with disabilities face bullying in school, discrimination in employment, and deprecation from family members. Some come to internalize these negative attitudes. In a comparative study of people with disabilities in Cameroon and India, researchers found that interviewees prioritized family members’ medical needs over their own impairments, which they grew to accept or treat with traditional remedies [14]. Even in cases such as people with disabilities in Kenya, Uganda, and Sierra Leone who succeeded in achieving economic independence, strategies revolved around self-advocacy and self-employment rather than community support [15].

## Existing Attempts to Expand Rehabilitation Services in LMIC

To respond to the urgent and unmet need for rehabilitation globally, the WHO announced a call to scale up rehabilitation efforts by 2030. As a response, several frameworks were formed including the Global Rehabilitation Alliance, the rehabilitation competency framework, and an update of the previously formed Community-Based Rehabilitation (CBR) framework. The Global Rehabilitation Alliance was formed in 2018 to supplement the development and delivery of rehabilitation universally. This alliance plays a key role in advocating for rehabilitation on a systemic level but remains advisory and does not address the competition for scarce resources in LMIC that limits rehabilitation services to people with disabilities, nor does it address obstacles at the professional or community levels.

Another response to the WHO call for action on the professional level has been the development of the rehabilitation competency framework. In 2018, a task force convened to organize a collection of competency statements for rehabilitation professionals to assist in overcoming workforce barriers. This framework (which is still under development) will assist rehabilitation practitioners worldwide by providing a unified language across disciplines while avoiding variation in terminology, concepts, and structure [16]. While this framework would assist in strengthening education and training, articulating standards of practice, and developing performance evaluation tools, it assumes an established base of rehabilitation services within health care systems. It does not address barriers on the professional level that impede referrals to therapeutic treatment.

Community-Based Rehabilitation (CBR) was established in 1978 by the WHO as an approach for social inclusion in resource-constrained settings and focused on working with people with disabilities within their communities. Since its establishment, CBR efforts have targeted barriers to access at the community level by equipping families, peers, and professionals as volunteers to deliver services without rehabilitation specialization [17]. Social outcomes and cost-effectiveness studies have shown positive emerging evidence of CBR as a service delivery approach in settings with a scarcity of resources [18]. CBR also shows promise in countering negative stigma about people with disabilities. However, the question of how it can be expanded to address barriers to rehabilitation at both the macrosystemic and professional levels remains largely unanswered.

## Piloting an Academic-Community Partnership

One promising approach that counters barriers at all three levels is to incorporate CBR with prelicensure health professions education. In 2017, the primary author launched Toward an All-Inclusive Jordan at MGH Institute of Health Professions, an independent, not-for-profit graduate school in Boston, Massachusetts, USA. The yearlong curriculum was designed to engage health professions students from the United States and Jordan in a CBR initiative in AlBaqaa refugee camp near Amman, Jordan, as a pilot. The program has three main components:

1. **Education:** MGH Institute graduate students in clinical doctorate programs in occupational and physical therapy and master’s students of speech-language pathology learn service delivery models of rehabilitation in LMICs. First, students complete a semester-long course in CBR and then conduct capstone graduation projects. Both elements include working in the field as rehabilitation trainers and providing direct service, including assessment, treatment planning, and rehabilitation intervention for people of disabilities and their families.
2. **Research:** Doctoral capstone projects of students are tailored to inform areas of rehabilitation and rehabilitation access in Jordan, such as studying the correlates of participation of children with disabilities in Jordanian schools and the prevalence of depression among Jordanians with physical disabilities.
3. **Advocacy:** Raising awareness about the rights of people with disabilities and educating community members about their legal rights is an integral component of the program. While they are visiting the refugee camp, graduate students help develop workshops to spread awareness among people of disabilities and their families about their rights. Additionally, the students work on developing content for social media platforms to address disability and stigma nationally.

**Figure 1:**
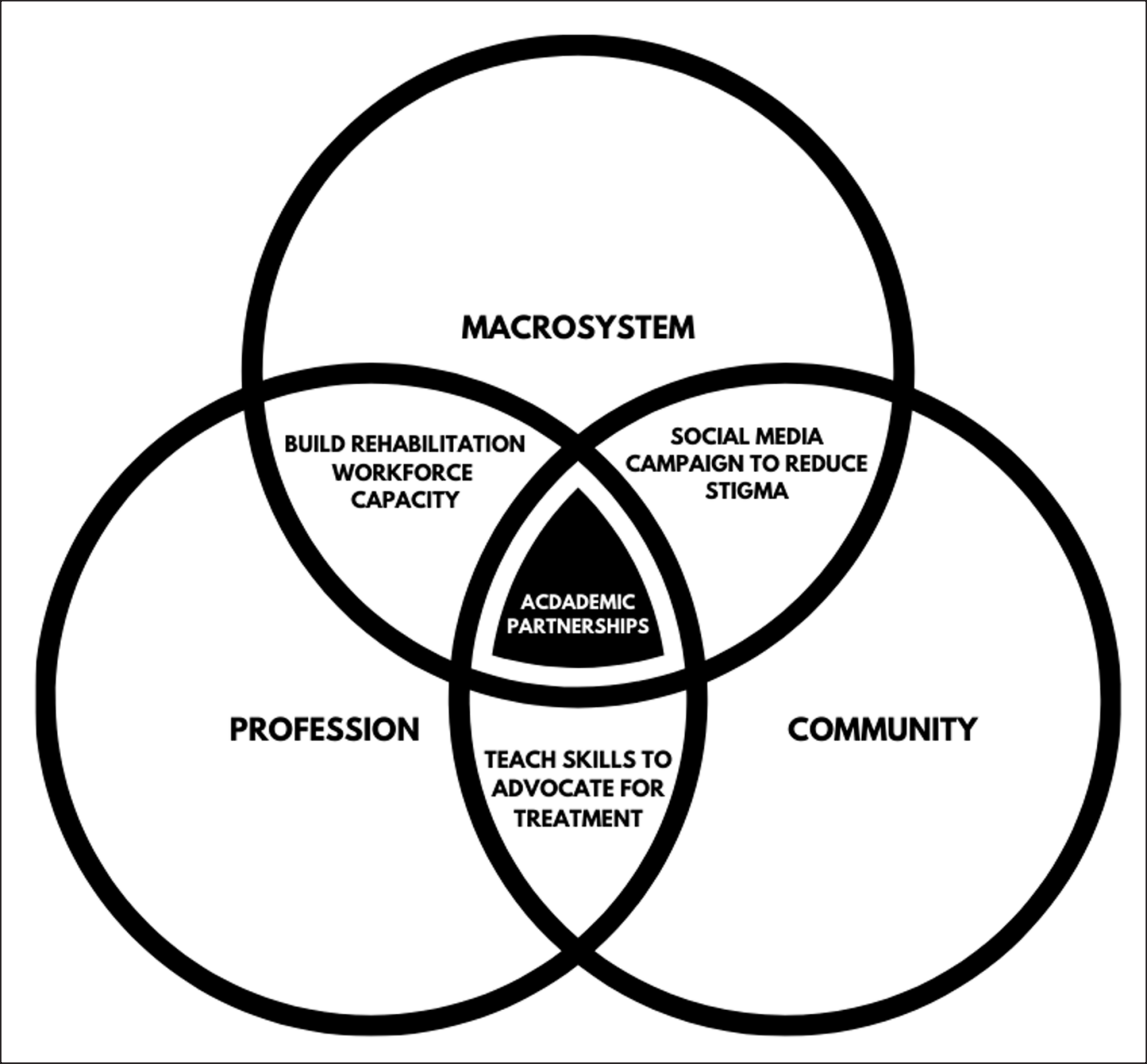
The CBR academic-community partnership addresses the primary barriers to expanding rehabilitation services.

The Toward an All-Inclusive Jordan approach counters the three levels of barriers to expanding rehabilitation services in LMIC (Figure 1). First, macrosystem partnerships were established in Jordan with the Jordanian Higher Council on the Rights of People with Disabilities and the higher coordination committee of the CBR centers for people with disabilities within the United Nations Relief and Works Agency for Palestine Refugees in the Near East (UNRWA). On a professional level, students from the MGH Institute partnered with students enrolled in occupational therapy, physical therapy, and speech-language pathology programs in Jordan to allow for capacity building of the local rehabilitation workforce. Partnerships were also established with local communities in refugee camps, where program activities were designed based on community needs. Community inclusion of people with disabilities was emphasized through social media campaigns and providing workshops to the community aiming to reduce stigma.

In two years, 14 Jordanian volunteers have partnered with 20 graduate students from MGH Institute of Health Professions to conduct over 300 direct rehabilitation sessions, ten workshops with over 100 mothers of children with disabilities (four of them livestreamed on social media with over 20,000 views), and trained over 12 CBR workers in refugee camps. To date, we are still in close contact with our collaborators in different refugee camps in Jordan. We continue to investigate methods of service delivery, and one idea we are piloting is the use of telehealth to support CBR workers.

The most noticeable long-term effect of our program was the fruitful collaborative partnerships established between American and Jordanian students and volunteers. Participants’ evaluations reflected that this experience nurtured action toward a meaningful shared cause, such as disability in this example. This common purpose could be the foundation for creating even more change on multiple levels. Some program alumni are already thinking of how they can transform rehabilitation systems through similar CBR programming in their home countries of Sri Lanka and Sudan.

The highest cost in running this program is hiring an on-site project manager who can coordinate logistics in the refugee camp. Students from the United States bear the costs of travel, accommodation, and tuition while in Jordan. While the example provided in this commentary is specific to our school, the many lessons learned can be applied to other settings and populations.

Despite the potential of this model, it has several limitations. First, faculty members planning global health experiences must rely on collaboration between departments, administrative leadership, and compliance offices. This work is usually not accounted for in the faculty members’ workload and might deter some from creating such opportunities in their institutions. A second limitation of community-based programs is the potential for political conflict or environmental concerns in the host country to interrupt travel from the high-income country, compromising the direct service model. In addition, communities in LMIC may not have the technological infrastructure to support telehealth arrangements. Finally, cultural differences can lead to differences in communication and execution patterns, but this, of course, can be a learning opportunity where the LMIC’s approach to problem-solving can be transferable to high-income countries.

## Conclusion

Expanding access to rehabilitation services in LMIC will require overcoming barriers on the macrosystemic, professional, and community levels. The academic-community partnership model builds on the proven success of community-based rehabilitation to provide basic rehabilitation services in LMIC while adding elements that address the competition for scarce resources and interprofessional hierarchies. Jordan, with its existing infrastructure of CBR, stakeholder buy-in, and identified need for services in refugee camps, has proven particularly fitting for an academic-community partnership. Each LMIC faces a different context, though the model of arraying challenges on three levels and tackling each of them through enhanced CBR applies broadly. The community-based rehabilitation component brings basic rehabilitation services to meet immediate needs and build advocacy skills. The academic component produces outcomes for research and interprofessional cooperation in a sustainable, cost-effective way. No country is immune from the tendency to favor acute medical interventions over long-term rehabilitative care. In this way, high-income countries can learn from the CBR efforts developed in LMIC to improve treatment for people with disabilities across the globe.
